# *PDSS1* mutations-associated steroid-resistant nephrotic syndrome: case report and review of literature

**DOI:** 10.1007/s00467-024-06596-y

**Published:** 2024-12-10

**Authors:** Clair Habib, Galit Tal, Karin Weiss, Daniella Magen, Shirley Pollack

**Affiliations:** 1 Clinical Genetics, Austin Health, Melbourne, Australia; 2 Metabolic Clinic, Ruth Rappaport Children’s Hospital, Rambam Health Care Campus, Haifa, Israel; 3 Rappaport Faculty of Medicine, Technion-Israel Institute of Technology, Haifa, Israel; 4 The Genetics Institute, Rambam Health Care Campus, Haifa, Israel; 5 Pediatric Nephrology Institute, Ruth Children’s Hospital, Rambam Health Care Campus, Haifa, Israel

**Keywords:** CoQ10 deficiency, Steroid-resistant nephrotic syndrome, Mitochondrial respiratory chain, Neuro-developmental delay

## Abstract

*PDSS1* mutations hamper Coenzyme Q10 biosynthesis and cause a rare multisystem mitochondrial disease characterized by diverse clinical features and limited treatment options. To date, renal involvement has been reported in only one patient. We report a new female patient with compound heterozygous *PDSS1* mutations and the clinical outcome following a trial of Coenzyme Q10 therapy. Our patient presented with developmental delay and regression at age three, which progressed to steroid-resistant nephrotic syndrome at age six, leading to stage 5 chronic kidney disease. Whole exome sequencing identified two pathogenic variants in the *PDSS1* gene. High doses of Coenzyme Q10 therapy had no effect at this advanced stage of disease. Coenzyme Q10 treatment did not appear to improve the clinical outcome in this patient. Further data is needed to better understand the phenotypic spectrum of *PDSS1-associated* disruption, and the potential benefit of early Coenzyme Q10 therapy.

## Introduction

Coenzyme Q10 (CoQ10) plays a critical role in various cellular processes, including ATP production via the mitochondrial respiratory chain. The biosynthesis of CoQ10 involves at least 15 genes, with at least ten of them linked to primary CoQ10 deficiency. The *PDSS1* gene encodes subunit 1 of decaprenyl diphosphate synthase, a key enzyme involved in the initial step of CoQ10 biosynthesis pathway [1].

Primary CoQ10 deficiency is an autosomal recessive disease with multisystem involvement and an age onset ranging from infancy to late adulthood. It is characterized by a wide spectrum of clinical features, ranging from isolated steroid-resistant nephrotic syndrome (SRNS) to a multisystem disease, which may include progressive cerebellar ataxia, intellectual disability, seizures, stroke-like episodes, neurosensory deafness, optic atrophy, cardiac involvement, and mitochondrial myopathy [2].

Treatment with CoQ10 supplementation in primary CoQ10 deficiency due to COQ2 mutations with isolated nephrotic syndrome has demonstrated promising results, including resolution of the nephrotic syndrome and restoration of normal kidney function [2]. We report a new female patient with CoQ10 deficiency due to compound heterozygous mutations in the *PDSS1* gene, who exhibited profound developmental regression accompanied by full-blown nephrotic syndrome. Following her genetic diagnosis, treatment with CoQ10 supplementation was initiated.

## Methods: patient description

This girl was the first-born child of non-consanguineous parents. She was born following an uneventful pregnancy. At 3 years of age, after experiencing an acute febrile illness accompanied by gastrointestinal symptoms, she was diagnosed with severe global developmental regression and epilepsy. Her metabolic workup demonstrated elevated blood levels of lactic acid at 1.8 mmol/L (normal values: 0–1.3 mmol/L), and cerebrospinal fluid alanine levels at 51 mmol/L (normal values: 16–41 mmol/L). Kidney function tests and urinalysis were unremarkable at that time. Coenzyme Q10 levels were not measured. Brain MRI showed bilateral symmetrical high-intensity white matter changes involving the basal ganglia, compatible with Leigh’s disease. Based on the clinical, biochemical, and imaging findings suggestive of a mitochondrial disorder, treatment with CoQ10 (2–8 mg/kg) was initiated as part of an empirical mitochondrial “vitamin cocktail”, combined with anti-epileptic therapy, although limited by poor parental compliance.

At the age of 6.5 years, following another acute febrile illness, she was diagnosed with nephrotic syndrome. Glomerular filtration rate was preserved, hypoalbuminemia of 1.6 g/dL, urinary protein to creatinine ratio of 20 mg/mg and systemic edema were the presenting clinical symptoms. Treatment with steroids was initiated but resulted in limited improvement. She was clinically classified as suffering from SRNS, leading to discontinuation of steroidal treatment. No further immunomodulating treatment was prescribed due to the suspicion of a mitochondrial disease, and a kidney biopsy was not performed due to parental refusal. She was treated with diuretics upon deterioration and exacerbated edema, while albumin infusions were not clinically indicated.

Whole exome sequencing was performed at the Genetic Institute of the Rambam Health Care Campus, revealing two likely pathogenic heterozygous mutations in the *PDSS1* gene: NM_014317.5: c.78del (p.Gly27Alafs*35) inherited from the mother, and NM_014317.5: c.637dup (p.Ser213Phefs*24) inherited from her father. Both mutations are truncating, causing a frameshift and premature stop codon, which are predicted to result in the loss of normal protein function. Given that the *PDSS1* gene is implicated in CoQ10 synthesis, treatment with high-dose CoQ10 (55 mg/kg/d) was resumed for nearly 2 years.

At the age of 8 years and 10 months, while on Q10 treatment, she developed an acute chronic renal failure, with elevated serum creatinine (11.7 mg/dL) and BUN (400 mg/dL), which was unresponsive to albumin or fluid infusion, resulting in hemodialysis dependence. Her clinical course was further complicated by intractable seizures, and she ultimately succumbed to her disease.

## Discussion

CoQ10 deficiency is an autosomal recessive disorder affecting the mitochondrial respiratory chain. CoQ10 is a vital component of this metabolic pathway, where it functions as an electron transporter essential for energy production in the body. The biosynthesis of CoQ10 primarily occurs in the mitochondria and involves several proteins. Mutations in any of the genes encoding those proteins (*PDSS1*, *PDSS2*, *COQ2*, *COQ4*, *COQ5*, *COQ6*, *COQ7*, *COQ8A/ADCK3*, *COQ8B/ADCK4*, *COQ9*) can potentially cause CoQ10 deficiency [2]. This disorder presents with a heterogeneous array of manifestations, affecting neurological development and function, and muscular and renal function. Specifically, *COQ2* mutations likely interfere with normal podocyte metabolism and impair mitochondrial biogenesis. While renal involvement, particularly SRNS, is a known manifestation of primary CoQ10 deficiency, mutations in *PDSS1* have thus far been reported in only a few patients with the multisystem phenotype of this disorder, while renal involvement was documented in only one patient [3]. Patients with *PDSS1* and *COQ2* mutations show the most severe clinical course and serious neurologic manifestations compared to other groups.

Patients with reported *PDSS1* mutations typically present with developmental delays and neurological symptoms. Most experience hearing loss, and ocular abnormalities are common. Some patients also exhibit hypotonia, while renal manifestations have been documented in only one case. These clinical features usually appear in early childhood, and death, reported in two patients, and also occurred during childhood.

To date, more than 60 patients with *COQ2* mutations have been reported, with phenotypes ranging from isolated nephrotic syndrome to multisystem disease. According to Drovandi et al., patients with *COQ2* mutations experienced the most frequent and severe extra renal manifestation compared to patients with variants in the *COQ6* and *COQ8B* genes. Of note, 20% of patients presented with disease onset during their third year of life, characterized by isolated SRNS without neurological manifestations [4].

The responsiveness to CoQ10 supplementation in CoQ10 deficiency has shown conflicting evidence. While some reports describe dramatic improvement in nephrotic syndrome and attenuation of renal disease progression with high-dose CoQ10 supplementation, others show less favorable outcomes. These findings underscore the importance of early genetic evaluation in cases of nephrotic syndrome. A mouse model harboring a *PDSS2* mutation leading to interstitial fibrosis, and a clinical phenotype of nephrotic syndrome progressing to kidney failure, demonstrated significant rescue with high-dose of CoQ10 supplementation [3]. Hence, treatment with high-dose CoQ10 (30–50 mg/kg/day) is recommended for CoQ10 biosynthesis defects, showing potential positive effect on the kidney function, though its benefits for other organ systems are limited [5]. A recent review by Drovandi et al*.* found that 56% in the global COQ2 cohort exhibited a significant reduction in proteinuria, with 6% achieving complete remission on COQ10 supplementation at doses of up to 60 mg/kg/day [6].

In two patients with mutations in *COQ2* gene, early treatment with CoQ10, initiated immediately after birth, led to the resolution of lactic acidosis and normalization of glucose levels in one patient, and improvement in proteinuria in the other. Although the treatment was believed to have a positive neurological effect, this benefit was only temporary, as both patients developed refractory seizures after 3 months of treatment, similar to their previously diagnosed siblings [7].

Three additional patients with *COQ2*-related disease, who presented with isolated SRNS, were treated with CoQ10 supplements. Two of them who began the treatment within the first 2 years of life showed improvement in both proteinuria and serum albumin levels. However, the third patient, who started treatment at the age of ten years, did not experience clinical improvement and ultimately required kidney replacement therapy. The lack of improvement in the last patient was postulated to result from low COQ10 levels within the renal tissue, despite normal serum levels [5].

A large cohort of patients with primary CoQ10 deficiency and glomerular involvement was described by Schijvens et al. [8]. Among this cohort, only one patient with a *PDSS1* mutation was reported. This patient presented with nephrotic syndrome, failure to thrive, and developmental delay at the age of 6 months. His condition rapidly progressed to kidney failure leading to his death at 16 months. The remaining patients in the cohort had mutations in other respiratory chain genes, including *PDSS2*, *COQ2*, *COQ6*, or *COQ8B/ADCK4* as outlined in Table 1, and were treated with CoQ10 with varying degrees of effectiveness [9].

**Table 1:** Literature published cases of CoQ10 deficiency and renal involvement

Journal	Article	Presentation	Treatment COQ10	Outcome
Pediatr Nephrol, 2018 [5]	COQ2 nephropathy: A treatable cause of nephrotic syndrome in children	3 patients with isolated steroid-resistant nephrotic syndrome	Patient 1 2 years old ubiquinone 30–50 mg/kg/day	Decreased proteinuria Improved serum albumin levels
			Patient 2 10 years old ubiquinone 30 mg/kg/day	No clinical improvement
			Patient 3: 2 years old ubiquinol 30–50 mg/kg/day	Urine protein to creatinine ratio improved
Pediatr Neurol, 2018 [9]	Response to early Coenzyme Q10 Supplementation is not sustained in CoQ10 deficiency caused by CoQ2 mutation	4 patients from 2 families with neonatal diabetes and proteinuria	Coenzyme Q10 treatment	Normalized glucose and proteinuria levels They developed refractory focal clonic seizures (at 3 months of age) that progressed to encephalopathy
Kidney Int Rep, 2020 [8]	Mitochondrial disease and the kidney with a special focus on CoQ10 deficiency	1 patient: *PDSS1* nephrotic syndrome, failure to thrive, developmental delay	Coenzyme Q10 treatment 30–50 mg/kg/day	Rapid renal failure and early death
		7 patients: *PDSS2*		50% no effect, 50% improved clinical condition
		28 patients: *COQ2*		14% neurological improvement, 29% neurological deterioration, 43% renal improvement, 29% no renal effect
		26 patients: *COQ6*		56% renal improvement, 83% hearing improvement
		82 patients: *COQ8B/ADCK4*		43% renal improvement, 57% no renal effect

*COQ* Coenzyme Q, *ADCK4*, AarF domain containing kinase-4

Most patients with *PDSS1* mutations reported from 2007 to 2022 (Table 2) did not receive COQ10 supplementation, with the exception of one case. This patient presented at 5 months of age with failure to thrive, hearing impairment, pulmonary hypertension, and distal phalangeal erythema. CoQ10 treatment was initiated at 15 mg/kg/day and increased to 30 mg/kg/day 3 months after diagnosis. Despite early intervention, the patient developed a progressive multisystem disorder, including chronic heart failure, and ultimately passed away at the age of three [10].

**Table 2:** Characteristics of previously published patients with *PDSS1* variants and this study

	Mollet et al. 2007 [7]		Vasta et al. 2012 [11]	Bellusci et al. 2021 [10]	Nardecchia et al. 2021 [1]		Jurkute et al. 2022 [12]						This study
PDSS1* variant*	Hom c.924T > G (p.Asp308Glu)	Hom c.924T > G (p.Asp308Glu)	c.661C > T (p.Arg221Ter) c.1108A > C (p.Ser370Arg	c.716 T > G (p.Val239Gly) c.1183C > T (p.Arg395*)	c.735G > T p.(Gln245His)	c.735G > T p.(Gln245His)	c.368 A > G p.(Glu123Gly) c.18 G > A p.(Trp6*)	c.232 C > A p.(His78Asn) c.886 G > A p.(Gly296Arg)	c.722–2 A > G p.(Ala204_Ala277del); p.(Gly241Alafs*6) c.589 A > G p.(Lys197Glu)	c.1118_1120dup p.(Val373_Gln374insLeu)	c.468–25 A > G p.? c.589 A > G p.(Lys197Glu)	c.893dup p.(Asn298Lysfs*11) c.589 A > G p.(Lys197Glu)	c.78del (p.Gly27Alafs*35) c.637dup (p.Ser213Phefs*24)
*Sex*	Male	Female	Female	Male	Female	Female	Female	Male	Female	Male	Male	Male	Female
*Age at report*	22	14	16 m	3	16	6	79	30	55	-	63	54	6.5
*Age of onset*	1	2	6 m	5 m	4	6	8	10	30	2	28	37	3
*Consanguinity*	Y	Y	-	N	Y	Y	N	N	N	N	N	N	N
*Ethnicity*	Moroccan	Moroccan	-	-	Egyptian	Egyptian	-	-	-	-	-	-	Korean
*Development*	Mild ID	Mild ID	GDD	Severe GDD	Mild ID	Mild ID	-	-	-	-	-	-	Severe GDD and regression
*Brain MRI*	-	-	Leukoencephalopathy/lactic acid peak on spectroscopy	-	Normal	-	-	-	-	-	-	-	Bilateral symmetrical high-intense white-matter changes involving the basal ganglia, compatible with Leigh’s disease
*Macrocephaly*	+	+	-	-	-	-	-	-	-	-	-	-	-
*Hypotonia/muscle weakness*	-	-	-	+	+	+	-	-	-	-	-	-	+
*Seizures*	-	-	-	+	-	-	-	-	-	-	-	-	+
*Hearing loss*	+	+	-	+	+	+	+	-	Mild	+	-	-	-
*Optic atrophy*	+	+	-	+	+	+	-	-	-	-	-	-	-
*Nystagmus*	-	-	-	+	-	-	-	-	-	-	-	-	-
*retinitis pigmentosa*	-	-	-	-	-	-	+	+	Retinal dystrophy	Retinal dystrophy	+	+	-
*Cataract*	-	-	-		-	-	-	-	Bilateral	-	+	-	-
*Cardiac*	Mild AR and MR, moderate PAH	-	-	Chronic heart failure	Moderate MR and TR,		Minor pericardial effusion	-	-	Bradycardia and syncope	MI	MI	-
*Failure to thrive/feeding difficulties*	-	-	+	+	-	-	-	-	-	-	-	-	-
*Peripheral neuropathy*	+	+	-	-	-	-	-	-	-	-	-	-	-
*Nephrotic syndrome*	-	-	+ acute tubular epithelial damage on biopsy	-	-	-	-	-	-	-	-	-	Steroid resistant nephrotic syndrome
*Livedo reticularis*	+	-	-	-	-	-	-	-	-	-	-	-	-
*distal phalangeal erythema*	-	-	-	-	-	-	-	-	-	-	-	-	-
*CoQ10 dosage*	-	-	-	15–30 mg/kg/day	-	-	-	-	-	-	-	-	2–55 mg/kg/day
*Outcome*			Died at 16 months due to rapid kidney failure	Died at age 3 years				-	-	-	-	-	Died at age of 6.5 years

*ID* intellectual disability, *AR* aortic regurgitation, *MR* mitral regurgitation, *TR* tricuspid regurgitation, *PAH* pulmonary artery hypertension, *GDD* global developmental delay, *MI* myocardial infarction

In conclusion, we report a young patient with a neurodevelopmental phenotype of *PDSS1*-related primary CoQ10 deficiency, who also developed SRNS that progressed to kidney failure and necessitated hemodialysis. Treatment with CoQ10 was initiated and gradually increased to a high dose, resulting in no significant improvement in either her renal or neurological condition. The compound heterozygous *PDSS1* mutations identified in our patient are predicted to have significant deleterious effects due to their truncating nature, leading to a severe deficiency of functional *PDSS1* protein. This impairs CoQ10 biosynthesis, resulting in a multisystem disorder with severe outcomes, including SRNS and neurological deficits. The lack of improvement with high-dose CoQ10 supplementation may be attributed to both the severity of the mutations and the delayed initiation of treatment. Additionally, although CoQ10 therapy was intermittently administered over the years, the initial dose was lower than recommended for CoQ biosynthesis defects. Early and continuous CoQ10 supplementation is generally considered more effective, and in this case, the delayed intervention may have been insufficient to prevent disease progression. Furthermore, as reported in the literature, all patients with *PDSS1* mutations showed minimal improvement with CoQ10 therapy, regardless of the timing of treatment initiation [7].

## Summary

### What is new?

- To the best of our knowledge, this is the second reported case of *PDSS1* mutations associated with primary CoQ10 deficiency and nephrotic syndrome. While some reports suggest that CoQ10 supplementation can improve renal disease, it appears to be ineffective in patients with this genetic defect. Both our patient and the previously reported case demonstrated rapid progression of renal disease, ultimately resulting in death, suggesting that *PDSS1* mutations may be linked to a more severe clinical presentation.

## Data Availability

All data supporting the findings of this study are available within the paper.
